# Missing Value Imputation of Time-Series Air-Quality Data via Deep Neural Networks

**DOI:** 10.3390/ijerph182212213

**Published:** 2021-11-20

**Authors:** Taesung Kim, Jinhee Kim, Wonho Yang, Hunjoo Lee, Jaegul Choo

**Affiliations:** 1 Kim Jaechul Graduate School of Artificial Intelligence, KAIST, Daehak-ro 291, Yuseong-gu, Daejeon 34141, Korea; zkm1989@kaist.ac.kr (T.K.); seharanul17@kaist.ac.kr (J.K.); 2 Department of Occupation Health, Daegu Catholic University, Gyeongbuk 38430, Korea; whyang@cu.ac.kr; 3 Department of Environmental Big Data, CHEM. I. NET, Ltd., Seoul 07964, Korea

**Keywords:** time-series data, spatio-temporal data, missing value imputation, interpretable deep learning, air pollution

## Abstract

To prevent severe air pollution, it is important to analyze time-series air quality data, but this is often challenging as the time-series data is usually partially missing, especially when it is collected from multiple locations simultaneously. To solve this problem, various deep-learning-based missing value imputation models have been proposed. However, often they are barely interpretable, which makes it difficult to analyze the imputed data. Thus, we propose a novel deep learning-based imputation model that achieves high interpretability as well as shows great performance in missing value imputation for spatio-temporal data. We verify the effectiveness of our method through quantitative and qualitative results on a publicly available air-quality dataset.

## 1. Introduction

Air pollution is one of the most challenging environmental problems attracting great global attention. It is the concentration of small harmful particles in the air, commonly caused by industry, power generation, and heavy traffic. Air pollution is a factor that greatly increases human mortality [1]. In 2015, 6.4 million people died because of polluted air worldwide; this is a much more significant number than those for AIDS (1.2 million), tuberculosis (1.1 million), and malaria (0.8 million) [2]. It shows the importance of preventing air pollution from getting worse.

Analyzing air quality data can be the first step in successfully preventing air pollution from getting worse and in forecasting future air quality. However, these data are often only partially observed, making it challenging to accurately analyze air quality. The hardware problem of air quality sensors and human error during data collection can lead to missing values. Moreover, since air quality data are collected from various locations simultaneously, missing values can very commonly occur. Many missing value imputation techniques have been studied to alleviate the missing value problem in collected data [3,4]. Especially, deep learning-based imputation methods using recurrent neural networks and generative adversarial networks have been studied and have achieved great success [5,6,7].

However, it is difficult to interpret the prediction results of these deep learning-based models due to the characteristic of data-driven algorithms. Recently, N-BEATS [8] has shown great success in time-series forecasting, as well as high interpretability. N-BEATS divides the prediction into three parts: trend, seasonality, and residual. By explicitly defining the trend and seasonality, N-BEATS enables us to study the reasons for prediction values. Inspired by these, we propose a novel deep learning-based imputation model that adopts the high interpretability of N-BEATS for the imputation task. Tailored to the missing value imputation task, our proposed model sequentially eliminates the bias, slope, seasonality, and residual of the input time-series, causing the output to become zero. Then, the summation of eliminated values can be used to represent the original time-series data. As our model uses explicitly defined bias, slope, and seasonality equations, the missing values can be imputed from them. Moreover, our model imputes missing values that occur in multiple locations simultaneously, utilizing the spatio-temporal information. To show the effectiveness of our method, we compare the proposed model with several commonly used imputation methods.

## 2. Materials and Methods

### 2.1. Datasets

This study is conducted using two different datasets: the (1) Guro-gu [9] and (2) Dangjin-si air quality datasets. A summary of the two datasets is provided in Table 1. We use PM_2.5_ and PM_10_ values for our experiments on both of the datasets. We split each dataset into two subsets according to a target variable, PM_2.5_ or PM_10_; in total, the four datasets are used for the experiments in this study.

The Guro-gu air quality dataset [9] is an air quality dataset collected from 24 different locations in Guro-gu, Seoul, South Korea. The data were collected every minute from 1 January 2020 to 31 July 2021. We utilize the data collected from 1 January 2021 to 31 July 2021 as test data. 80% of the remaining data are used as training data and 20% are used as validation data. We can not obtain the ground truth values for missing values in real datasets. Thus, for evaluation of the missing value imputation performance, we additionally make 20% of the test data missing and then measure the model performance on them. The values are removed completely at random. As 7.91% of the test data are missing in a natural situation, 26.32% of the data are missing when the additional missing values are considered.

The Dangjin-si air quality dataset is an air quality dataset collected from 42 different locations in Dangjin-si, Chungcheongnam-do, South Korea. The data were collected every minute from 28 May 2020 to 31 July 2021. The data collected from 1 January 2021 to 31 July 2021 are used as test data. 80% and 20% of the data collected from 28 May 2020 to 31 December 2020 are used as training data and validation data, respectively. As in the Guro-gu air quality dataset, we eliminate 20% of the test data values and evaluate the imputation performance for the eliminated values. The missing rates of test data before the elimination and after the elimination are 16.1% and 32.9%, respectively.

### 2.2. Imputation Method

**Figure 1:**
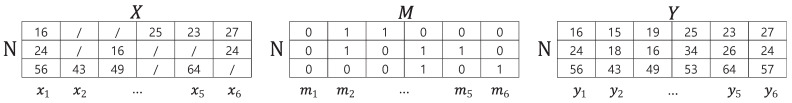
Example of time-series data with missing values X, its corresponding missing value mask M, and time-series data without missing values Y. The slash (/) denotes the missing values. $x_{i}\in R^{N}$ is the *i*-th observation of the target variable collected from *N* different locations, where N=3 for this example.

We consider the spatio-temporal imputation task. Given a time-series matrix $X=\{x_{1},x_{2},\cdots ,x_{T}\}$ with missing values and a missing value mask matrix $M=\{m_{1},m_{2},\cdots ,m_{T}\}$, where *T* denotes the length of a input sequence, $x_{t}\in R^{N}$ denotes the *t*-th observation of X, and *N* denotes the number of locations of data collection, we aim to predict the time-series data without missing values $Y=\{y_{1},y_{2},\cdots ,y_{T}\}$. The *n*-th feature of the input data represents the time-series collected from the *n*-th location of data collection. Figure 1 shows examples of X, M, and Y.

**Figure 2:**
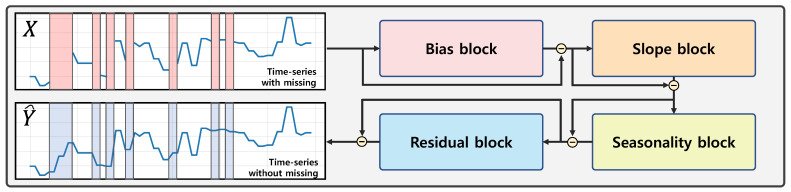
Overview of proposed model. X and $\hat{Y}$ indicate the input and output time-series of the model, respectively.

As shown in Figure 2, our model consists of four different blocks: a bias block, slope block, seasonality block, and residual block. The blocks sequentially eliminate the bias, slope, seasonality, and remaining part of the input time-series data.

At first, the bias block estimates the average value of the non-missing values $h^{\mathrm{bias}}$ as

(1) $$\begin{array}{c} h^{\mathrm{bias}}=h^{\left(1\right)}=\frac{\sum_{i=1}^{T}x_{i}m_{i}}{\sum_{i=1}^{T}m_{i}}. \end{array}$$

After that, the output of the bias block is calculated as $X^{\left(1\right)}=X-h^{\left(1\right)}$. Then, the rest of the blocks, i.e., slope, seasonality, and residual blocks, compute the outputs as follows: considering the *l*-th block, it encodes the output of the previous block $X^{\left(l-1\right)}$ into a coefficient $\theta^{\left(l\right)}$ using five fully connected layers with LeakyReLU non-linearity [10] as

(2) $$\begin{array}{c} \theta^{\left(l\right)}={\mathrm{FC}}_{5}^{\left(l\right)}\left(\sigma \left({\mathrm{FC}}_{4}^{\left(l\right)}\left(\sigma \left({\mathrm{FC}}_{3}^{\left(l\right)}\left(\sigma \left({\mathrm{FC}}_{2}^{\left(l\right)}\left(\sigma \left({\mathrm{FC}}_{1}^{\left(l\right)}\left(\mathrm{Flatten}\left(X^{\left(l-1\right)}\right)\right)\right)\right)\right)\right)\right)\right)\right)\right), \end{array}$$

where FC denotes the fully connected layer and σ denotes the non-linearity. A coefficient $\theta^{l}$ is a scalar for the slope block, eight-dimensional vector for the seasonality, and 256-dimensional vector for the residual block. After obtaining $\theta^{\left(l\right)}$, it is used as a coefficient for the specific function depending on the block type. The equations of the slope $h^{\mathrm{slope}}$, seasonality $h^{\mathrm{seasonality}}$, and residual $h^{\mathrm{residual}}$ are

(3) $$\begin{array}{c} h^{\mathrm{slope}}=h^{\left(2\right)}=\theta^{\left(2\right)}v, \end{array}$$

(4) $$\begin{array}{c} h^{\mathrm{seasonality}}=h^{\left(3\right)}=\sum_{i=1}^{4}\theta_{2i}^{\left(3\right)}\sin\left(2\pi iv\right)+\theta_{2i+1}^{\left(3\right)}\cos\left(2\pi iv\right), \end{array}$$

(5) $$\begin{array}{c} \mathrm{and}\;h^{\mathrm{residual}}=h^{\left(4\right)}={\mathrm{FC}}_{6}^{l}\left(\sigma \left(\theta^{\left(4\right)}\right)\right), \end{array}$$

where v=[−T/2,−T/2+1,⋯,0,⋯,T/2−1] is the vector denoting the time horizon of the input time-series and ${\mathrm{FC}}_{6}^{l}$ is another fully connected layer. Finally, the output of the *l*-th block is calculated as

(6) $$\begin{array}{c} X_{l}=X_{l-1}-h^{\left(l\right)}. \end{array}$$

As we eliminate the bias, slope, seasonality, and residual of the input time-series data, the final model output $X_{l}$ should become a matrix with zero values. In other words, the summation of $h^{l}$ should become the original data. With this in mind, we train the model to minimize the mean absolute error between the summation of eliminated values $\hat{Y}=\sum_{l=1}^{4}h^{\left(l\right)}=h^{\mathrm{bias}}+h^{\mathrm{slope}}+h^{\mathrm{seasonality}}+h^{\mathrm{residual}}$ and the ground truth values without missing Y. In the inference phase, we use $\hat{Y}$ as a final imputation result.

For the slope block, we use the output of the FCs (Equation (2)) as the inclination of the linear function without bias (y=ax). Therefore, the output of the FCs should represent the inclination coefficient of the input time-series to minimize the prediction error. Similarly, the output of the FCs in the seasonality block is used to represent the coefficient of the cosine and sine functions (the coefficient of the Fourier series). Therefore, the final output of the seasonality block is also periodic. To minimize the imputation error, the FCs in the seasonality block should capture the appropriate coefficient of the periodic function to represent the seasonality in an input time-series.

Additionally, when training, we can not obtain the ground truth values for missing values in real datasets. Therefore, we use an additional technique to effectively train the model to impute the missing values. Following the previous work [11], we additionally drop the part of input data randomly for every iteration, and train the model to restore the dropped values. During the training, we use the time-series with 20% of additional missing values as an input time-series X and use the original time-series as a ground truth time-series Y. The model is trained to minimize the mean absolute error for the non-missing values of Y.

### 2.3. Experimental Details

For the training of the proposed model, we utilize Adam optimizer [12] with hyperparameters $\beta_{1}=0.9$ and $\beta_{2}=0.999$. The output hidden vector dimension of fully connected layers in the blocks is set to 64. We chose the vector dimensions of $\theta^{\left(l\right)}$ of the seasonality and residual blocks as empirically showing the best performances. The input horizon is set to 60, so that the model imputes the one-hour data at once. The negative slope of LeakyReLU non-linearity is set to 0.2. During the training, we shift up the input data with a value from zero to ten with a probability of 25% and scale the input data with a factor from zero to three with a probability of 25%. The shifting and scaling can be applied simultaneously. We set the batch size to 512 and the learning rate to 0.00001. We train the model until there is no performance improvement on the validation dataset for 5 epochs. An Intel^®^ Xeon^®^ Processor E5-2650 v4 machine equipped with 128GB RAM is used to conduct the experiments. The models are trained on a single NVIDIA Titan X GPU with a random seed 42 in an Ubuntu 16.04.6 LTS environment. All the experiments are implemented in the PyTorch 1.7.0 deep learning framework [13] using Python 3.6.10.

### 2.4. Evaluation Metric

We measure the model performance with two metrics: mean absolute error (MAE) and symmetric mean absolute percentage error (sMAPE). MAE is a common evaluation metric for time-series imputation calculated as $\mathrm{MAE}=\frac{1}{B}\sum_{i=1}^{B}\left|\hat{y}-y\right|$ where *B* denotes the number of input data. However, since it averages out the error without considering the scale of the error, it can be inaccurate when the error scale changes over time. In contrast, sMAPE, which is calculated as $\mathrm{sMAPE}=\frac{1}{B}\sum_{i=1}^{B}\frac{|\hat{y}-y|}{(\hat{y}+y)/2}\cdot 100\%$, is a scale-invariant metric. Considering that the values of PM_2.5_ and PM_10_ vary from zero to over a thousand, the scale-invariant metric can accurately measure the imputation performance of the model. Moreover, even when an observed value is zero, sMAPE can be utilized, in contrast to the mean absolute percentage error, $\mathrm{MAPE}=\frac{1}{B}\sum_{i=1}^{B}\frac{|\hat{y}-y|}{y}\cdot 100\%$, one of the commonly used scale-invariant metrics.

### 2.5. Baseline Models

We compare the proposed model with the following baselines:

Mean substitution (Mean): The missing values are substituted with the average value of the training dataset.
Spatial average value substitution (SA): We replace the missing values with the average value of the data collected from different locations. The value is calculated as $\hat{y_{i}}=\frac{1}{N}\sum_{j=1}^{N}x_{i}^{j}\left(1-m_{i}^{j}\right)$, where $x_{i}^{j}$ indicates the input data at time step *i* that is collected at the *j*-th data collection location.
Multivariate imputation by chained equations (MICE): We use MICE [3] to impute the missing values. MICE makes multiple imputations using chained equations. MICE is implemented using the FancyImpute library.

## 3. Results

We cannot obtain the complete real datasets. Therefore, we additionally eliminate 20% of the test datasets and measure the imputation error on them. The eliminated values are unseen data for the model and used only for the evaluation of the imputation performance. The imputation error is measured using MAE and sMAPE. Table 2 shows the imputation performance for the proposed model and the baselines, on Guro-gu air quality dataset. As shown in the table, mean imputation is very inaccurate. The average value of the data collected from different locations shows a better performance than the mean imputation. MICE surpasses the mean and spatial average value imputation methods. However, it still has significantly large error, especially for PM_10_ (9.291 MAE and 31.408 sMAPE). The proposed model consistently outperforms the baselines by a large margin.

Table 3 shows the imputation error on the Dangjin-si air quality dataset. Even when performance is evaluated with the data measured in Dangjin-si, a tendency similar to that of the results of the Guro-gu data appears. Simple naive imputation methods, i.e., the mean imputation method and the spatial average value substitution method, show large errors compared to MICE and our proposed model. The proposed model shows much smaller error than MICE.

**Figure 3:**
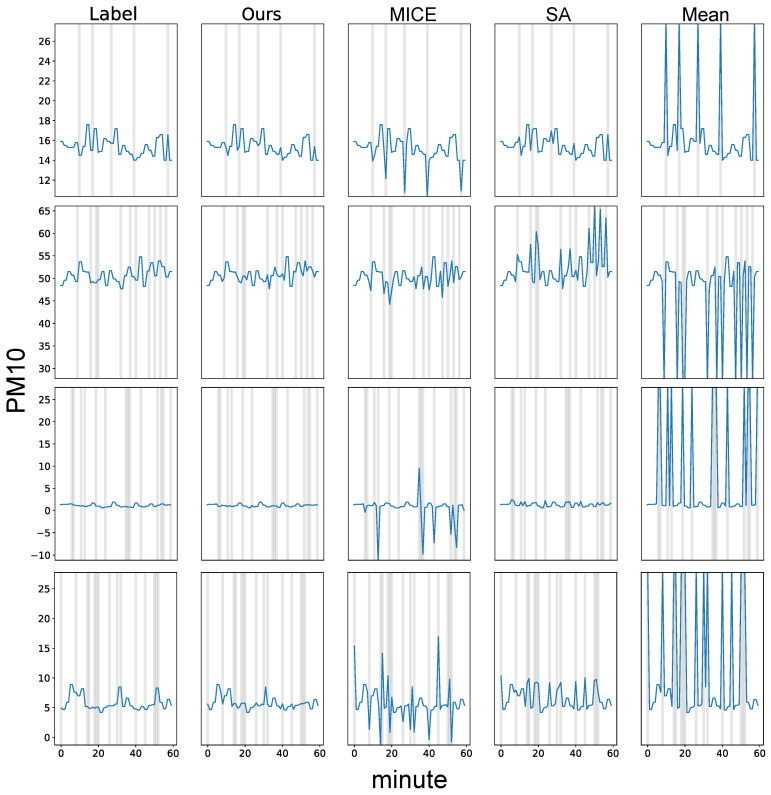
Qualitative time-series imputation results of our method in comparison with baseline models. The results are obtained with PM_10_ test data collected at Guro-gu. We mark the missing values in the input as gray background. For all the compared models, the non-missing values (white background) are the same as the label. Label denotes the original time-series without missing values. SA indicates the spatial average value imputation method.

To further study the effectiveness of our proposed model, we illustrate the prediction results in Figure 3. Our method consistently shows the results most similar to those of the label. MICE and spatial average value substitution methods show competent results. However, they failed to accurately predict all missing values. The mean imputation method does not capture the input time-series information, leading to poor imputation performance.

**Figure 4:**
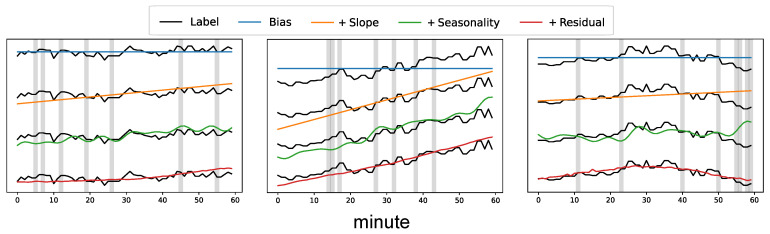
Cumulative prediction results of the bias, slope, seasonality, and residual blocks. The results are obtained with PM_2.5_ test data collected at Guro-gu. Label denotes the original time-series without missing values.

Figure 4 illustrates the interpretability of our proposed model. The cumulative prediction results of the bias (blue line), slope (orange line), seasonality (green line), and residual (red line) blocks are shown in the figure. To compare each prediction result with the label (black line), we draw the label four times. We use the PM_2.5_ test data collected at Guro-gu. In real application, the missing values (gray background) are imputed with the final model prediction (red line).

## 4. Discussion

Several studies have used deep learning based models to impute missing values of time-series data. For example, Che et al. [5] proposed a recurrent neural network based missing value imputation method. It utilizes the time interval that contains information on how long the values have not been observed, so that the model can choose whether to use the information of last observed value automatically. This study highlighted the potential of deep learning-based imputation methods. Luo et al. [6] used a generative adversarial network to generate the missing values. It significantly improved the imputation performance but had limited real application because the additional training procedure is included in the inference phase, leading to slow inference speed. Cao et al. [14] proposed a bidirectional recurrent neural network for imputation of time-series data. They showed the effectiveness of their imputation method with the application of imputed data to classification tasks.

Compared to previous studies, we try to explicitly express the time-series data in terms of bias, slope and seasonality, so that we can interpret the prediction results of the model by dividing them into trend, seasonality, and residual. By doing so, we achieve competent imputation performance, surpassing those of mean imputation, spatial average value imputation, and MICE by a large margin. The qualitative imputation results also show the effectiveness of our method. It is notable that our method consistently predicts a similar results to those of the groundtruth. Additionally, cumulative prediction results of the bias, slope, seasonality, and residual blocks show the interpretability of our model prediction.

However, our method has a limitation. The prediction result of the seasonality block is not fit well to the original value. The poor performance of the seasonality block mainly comes from the Fourier function that represents the seasonality in an input time-series. We used a finite discrete Fourier series with pre-defined periods, and consequently, the model can not capture the seasonalities having a different period from the pre-defined ones. In addition, the seasonalities of the Guro-gu and Dangjin-si datasets appear in quite long periods, e.g., yearly basis, which is difficult for the model to handle at once due to the limitation of computational resources. We will find the appropriate seasonality function for air quality data in future work. Utilizing our model has the advantage of allowing us to know the problem of the model through interpretable prediction results.

## 5. Conclusions

This paper proposes a novel end-to-end model that imputes missing values in air quality time-series data. The model predicts the bias, slope, seasonality and residual of an input time-series data, so that missing values can be imputed by combining them. Our method surpasses several commonly used imputation methods, e.g., mean imputation, spatial average value imputation, and MICE at imputing missing values in the Guro-gu and Dangjin-si air quality datasets. Qualitative results comparing the proposed method and the baselines show the effectiveness of our method.

## Figures and Tables

**Table 1:** Summarization of two air quality datasets. We report the statistics with the whole dataset including training data, validation data and test data. Stdev. denotes the standard deviation of the data. # locations indicates the number of data collection locations.

Dataset	Mean	Stdev.	# Observations	# Locations	Missing Rate (%)
Guro-gu (PM_2.5_)	21.931	30.593	827,051	24	26.014
Guro-gu (PM_10_)	34.275	47.650	827,049	24	26.027
Dangjin-si (PM_2.5_)	24.916	41.423	464,720	42	28.964
Dangjin-si (PM_10_)	43.914	190.288	464,720	42	28.963

**Table 2:** Imputation performances of the proposed method and of other imputation methods on the Guro-gu dataset. Our results show the performance of the proposed method, which achieves the best imputation accuracy.

Target Variable	Metric	Ours	Mean	SA	MICE
PM_2.5_	MAE	1.170	18.634	8.972	4.825
	sMAPE	7.155	75.236	36.771	28.865
PM_10_	MAE	2.738	30.024	17.646	9.291
	sMAPE	9.385	73.259	43.464	31.408

**Table 3:** Imputation performances of proposed method and other imputation methods on the Dangjin-si dataset. The proposed method shows the best imputation accuracy.

Target Variable	Metric	Ours	Mean	SA	MICE
PM_2.5_	MAE	1.149	16.780	9.646	4.524
	sMAPE	9.710	81.604	52.389	34.859
PM_10_	MAE	4.664	33.521	20.465	12.279
	sMAPE	13.702	86.151	56.168	44.624

## Data Availability

Data available on request due to restrictions. The data presented in this study are available on request from the corresponding author. The data are not publicly available since the permission for use by the Ministry of Environment, Guro-gu, and Dangjin-si is required.

## Abbreviations

The following abbreviations are used in this manuscript:

MAE	Mean absolute error
sMAPE	Symmetric mean absolute percentage error
